# Cytidine deamination-induced perpetual immunity to SAR-CoV-2 infection is a potential new therapeutic target

**DOI:** 10.7150/ijms.61779

**Published:** 2021-10-15

**Authors:** Asad Ullah, Neelam Mabood, Muhammad Maqbool, Luqman Khan, Mujib Ullah

**Affiliations:** 1 Department of Radiation Oncology, School of Medicine, University of California San Francisco, San Francisco, California, USA.; 2 Department of Pediatrics, University of Alberta, Edmonton, Alberta, Canada.; 3 Department of Clinical & Diagnostic Sciences, The University of Alabama at Birmingham, Birmingham, AL, USA.; 4 Cardiovascular Research Institute, School of Medicine, University of California San Francisco, San Francisco, California, USA.; 5 Department of Immunology and Transplantation, School of Medicine, Stanford University, Stanford, California, USA.

**Keywords:** Perpetual immunity, Cytidine deaminase, SRS-CoV-2, New variants of Coronavirus

## Abstract

As the world is racing to develop perpetual immunity to the SARS-CoV-2 virus. The emergence of new viral strains, together with vaccination and reinfections, are all contributing to a long-term immunity against the deadly virus that has taken over the world since its introduction to humans in late December 2019. The discovery that more than 95 percent of people who recovered from COVID-19 had long-lasting immunity and that asymptomatic people have a different immune response to SARS-CoV-2 than symptomatic people has shifted attention to how our immune system initiates such diverse responses. These findings have provided reason to believe that SARS-CoV-2 days are numbered. Hundreds of research papers have been published on the causes of long-lasting immune responses and variations in the numbers of different immune cell types in COVID 19 survivors, but the main reason of these differences has still not been adequately identified. In this article, we focus on the activation-induced cytidine deaminase (AID), which initiates molecular processes that allow our immune system to generate antibodies against SARS-CoV-2. To establish lasting immunity to SARS-CoV-2, we suggest that AID could be the key to unlocking it.

## Introduction

The year 2021 will be remembered as a time when the world was dealing with new variants of the acute respiratory syndrome coronavirus (SAR-CoV-2) and celebrating the phasing out of vaccines to combat the virus. Humanity has suffered tremendously as a result of SARS-CoV-2 outbreaks, but this has not dampened their determination, which has been tested in the adoption and response to a global COVID-19 pandemic. The emergence of new strains of COVID-19 in various parts of the world has created ripples and caused researchers to adopt different approaches to deal with the new strains of SARS-CoV-2 1. We were fortunate that the vaccine against COVID-19 had been discovered in less than a year, which would otherwise have taken years to succeed. To illustrate, it took years for the mumps vaccine to be produced and, historically, it was the quickest discovery before the breakthrough of the COVID-19 vaccine later in 2020 2, 3. The discovery no doubt was the culmination of years of diligent work by researchers across the globe. Since the emergence of coronaviruses such as SARS and MERS, the scientific community has begun this initiative. The latest data on the COVID-19 indicates that only 17% of people infected with SARS-CoV-2 show no symptoms and these individuals are 42% less likely to transmit the virus 4, 5. People wonder why certain individuals are asymptomatic despite being infected with the virus. The most likely response to this question is that asymptomatic people have a different immune response to the virus. Interestingly, a study conducted by Xin Zhang of Huazhong University of Science and Technology does provide a response to our question. According to the study, the antiviral effects of antibodies outweigh any potential negative effects, at least during the early stages of COVID-19 6. Another study suggested that asymptomatic individuals have roughly one fourth the chance of spreading infection and that they are infectious for a shorter time 7. Thus, it is important to recognize the immune response that safeguards asymptomatic individuals against infection with COVID 19 viruses. While conflicting data has been recorded through publications on the infectivity of asymptomatic infection, there isn't a single publication that explains the possible underlying mechanism that enables asymptomatic carriers to get through COVID-19 without displaying typical symptoms and recover quickly. This inquiry may lead us to the previously unknown domain of perpetual immunity against SARS-CoV-2. In this article, we explore the possible molecular mechanism by which asymptomatic SARS-CoV-2 patients develop a different level of immunity. We posit that activation-induced cytidine deaminase (AID), which in normal circumstances is catalytically inefficient, improve antibody affinity by deaminating cytosines within immunoglobulins (Ig) hence potentiating the humoral response against SARS-CoV-2 immunogenicity 8. AID is the protein that initiates the first molecular processes to antiviral immunity 9-11. As with the growing infection and emerging new COVID-19 variants, we assert that probing new ways to design vaccines, therapeutics, or agonist agents is the need of the time. As the vaccination drive is in full swing, the worry of reinfection or getting infected after vaccination is hovering our minds. With so much uncertainty, we must recognize that our bodies' immune defenses are designed to produce effective antibodies against a specific invader. Our immune system makes a recollection of its previous experience with pathogens. Upon reinfection with the same invader, our immune system robustly identifies the invader and protects against it 12. Regarding coronavirus infection, we have instances where the reinfection did not cause any symptoms but there are cases where the second infection was devastating 13, 14. For now, we have no evidence that a single individual has been reinfected for the third time. With our current immunology knowledge, we can predict that a third re-infection, if it occurs, will aid in the development of long-term immunity, leading to perpetual immunity. Is this a novel way of looking at immunity? No, absolutely not. The innate immune system is an evolutionary conserved host defense system that can sense the invading antigen. As suggested initially that SARS-CoV-2 mutates far less than HIV or influenza viruses, it is now clear that SARS-CoV-2 variants (D641G) could be sporadic 15. The fact that the new strains are sporadic does not imply that they are more infectious; rather, these new variants may cause the evolution of our immune system. We anticipate that our immune system will adapt to the virus and will produce a diverse repertoire of immunoglobulins (Ig) which have stronger affinity to knock down the virus. Studies have shown that activation induced cytidine deaminase (AID) activity could provide answers to such evolving strategies as it has been involved in initiating programmed DNA alterations leading to antibody diversification for countering infinite number of infections causing agents 16, 17. Although recent reports indicate that the antibody titer of asymptomatic people with SARS-CoV-2 is different from that of symptomatic patients and that the humoral response to the virus diminishes rapidly in patients with no symptoms 18-20. We believe this does not apply universally, as shown by studies suggesting that the longevity of the immune response to the virus is growing. Latest data implies that antibodies in COVID-19 stabilized patients will survive up to eight months, while previous literature predicted they will disappear in three months from the time the patients first experienced symptoms 21-23. We assume as more people are infected with either new strains or older forms of the virus, and more and more people are constantly vaccinated will help people develop a humoral response that will forever outperform the SARS-CoV-2 virus. In addition, findings have demonstrated that despite the fact that both symptomatic and asymptomatic patients have comparable viral loads from their throat swab, there is clear indication that the virus is resolved more rapidly in patients with no symptoms. These observations lend credence to the notion that the immune system of asymptomatic people can neutralize the virus differently.

We speculate that in asymptomatic patients, AID is preferentially activated, fostering an efficient humoral immune response by altering the immunoglobulin genes (Ig) that code for antibody. Reinfections, vaccination, and the persistent appearance of new SARS-CoV-2 strains would put AID to the test in terms of producing antibodies that would forever neutralize the virus. Antibody maturation by AID may be the basis for lifelong immunity to the virus, and this is where researchers can focus their efforts as a possible novel therapeutic target.

## Is AID a gateway to a strong and enduring immune response?

**Figure 1:**
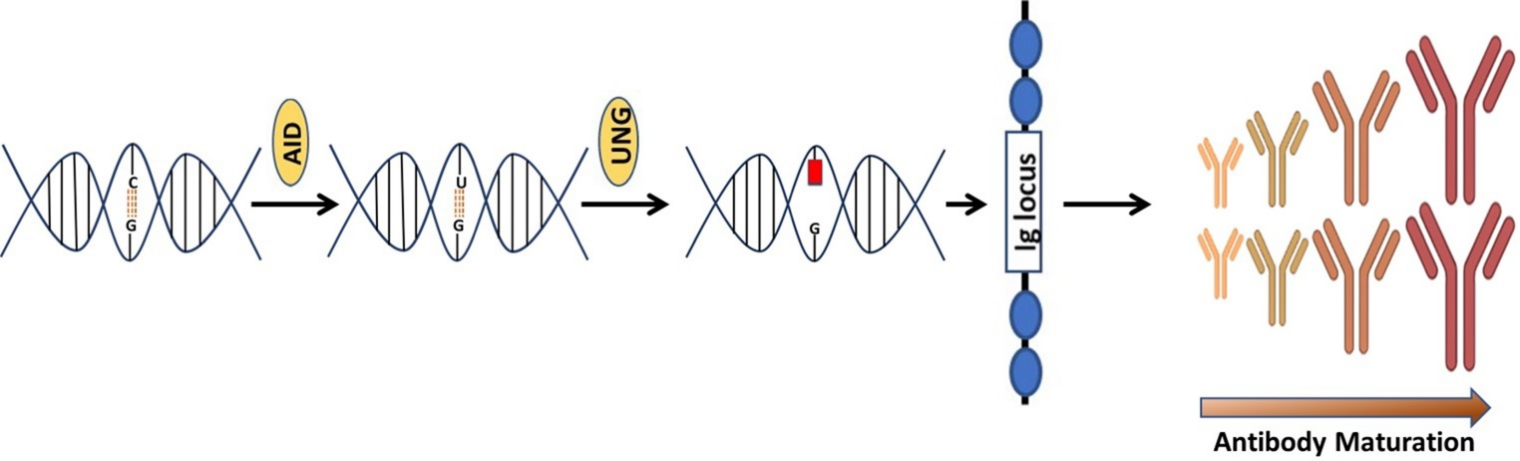
** An illustration that how AID is involved in the generation of antibodies.** Consequences of Cytosine (C) deamination by activation induced cytidine deaminase (AID). AID remove amino group 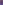 from C to produce uracil (U). Upon interception by uracil DNA glycosylase, an abasic site is created causing SHM & SRC of Ig genes.

Discovered by Tasuku Honjo and associates in 1999 24, AID is an enzyme that is linked with the process of antibodies diversification**.** In vertebrates, primary antibodies are generated by site-specific recombination of variable (V), diversity (D) and junction (J) segments during B cell development. The process of antibody diversification begins when a patient experiences an antigen for the first time. This association of the antibody with the invasive antigen not only triggers the killing of the infectious organism by other cells of the immune system, it also enhances its ability to resist infections successfully in subsequent interactions with the same antigen. Mechanistically, AID begins the process by catalyzing the deamination of cytosine, which results in a uracil: guanine (U:G) mispair 25-28. The mispair is further processed by uracil DNA glycosylase (UNG) creating an abasic site. Translesion polymerases replicate over the abasic site generate mutations causing class switch recombination (CSR) or somatic hypermutation (SHM) **(Figure 1)**. These genetic alterations produce antibodies with greater affinity to the specific antigen. A new population of antibodies with a higher level of fitness to combat an infectious agent is produced through a clonal selection process 29, 30.

**Figure 2:**
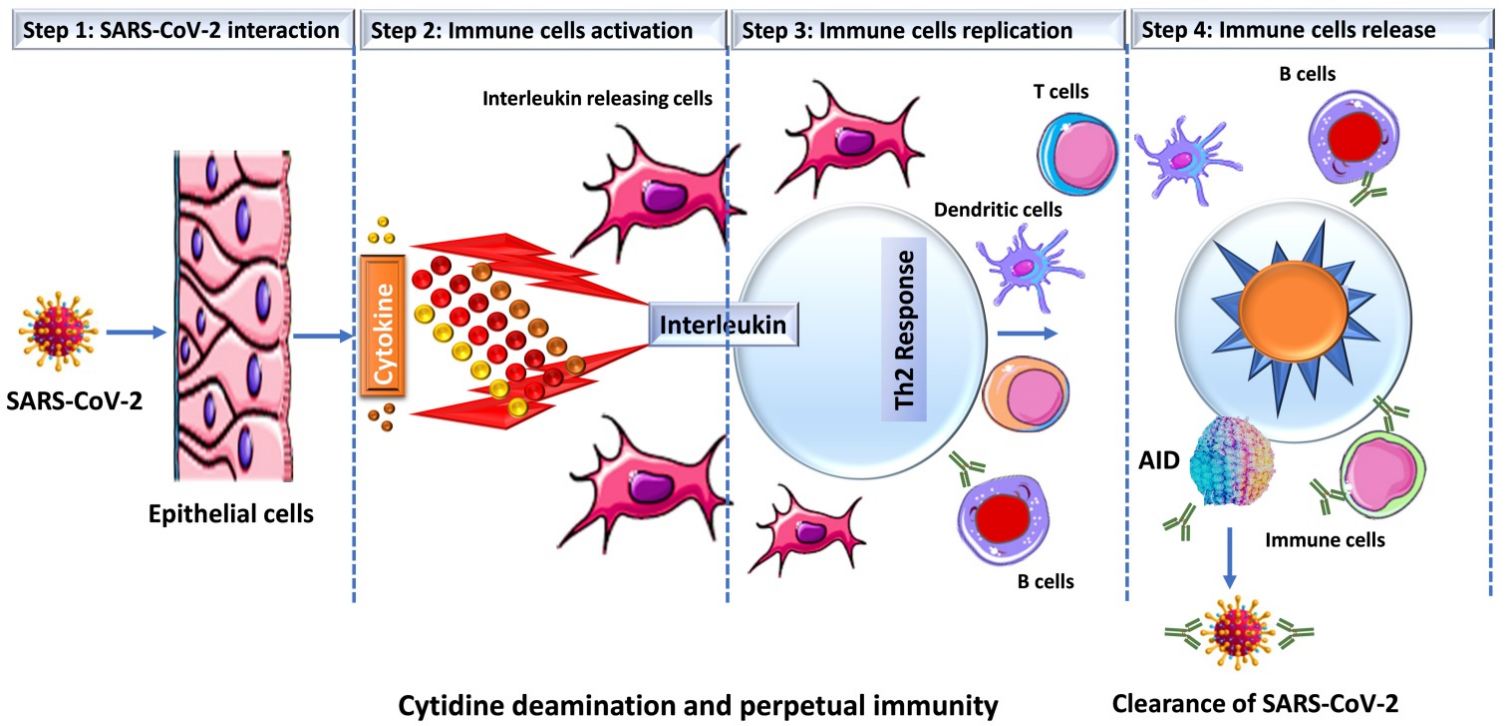
** Schematic representation of SARS-CoV-2 infection and cytokine release.** Once SARS-CoV-2 infect lung epithelial cells, the immune system produces cytokines that control the NFkB pathway, which is activated by interleukins (IL4 or IL6) and results in an increase in the expression of activation-induced cytidine deaminase (AID) and apolipoprotein B mRNA-editing enzyme, catalytic polypeptide (ABOPEC) genes. Overexpression of AID results in the development of high affinity antibodies that destroy the virus.

Prior studies have suggested a strong correlation between AID induction and *in vivo* antibody affinity maturation in humans 31, 32. Based on these results, we hypothesize that SARS-CoV-2 infection produces a microenvironment conducive to efficient AID expression, resulting in an immune response strong enough to keep an infected person asymptomatic while also establishing long-term immunity. When an individual is infected with SARS-CoV-2, a conglomerate of factors begins to shape the microenvironment that possibly enhances the expression of AID and initiate the process for antibody maturation. This includes an overactive Th2 response with elevated IL-4 levels that is bolstered even more by a hike in CD40 ligand (CD40L) level 33-35. In B cells, both of these factors activate cell proliferation, Ig switching and antibody production 36, 37. Studies have shown that when CD40L binds to its receptor (CD40) on B cells, it triggers a signal that synergize with the signal produced by the IL4's interaction with its receptor, resulting in an optimal AID gene expression in both human and mouse B cells 38-42. This is how B cells produce a wide range of antibodies with a higher affinity for the virus that is targeting them. In addition, evidence indicates that IL4 and CD40L alone can trigger AID mRNA expression 43, 44. As shown in **Figure 2**, that SARS-CoV-2 infection triggers the release of pro-inflammatory cytokines (interleukins), which boosts the expression of APOBEC and AID through the NFkB pathway. The upregulation of AID leads to the production of high affinity antibodies, which assist in the removal of the virus while also setting the foundation for long-term immunity. AID is a member of the apolipoprotein B RNA-editing catalytic component (APOBEC) family of enzymes that transform cytosines (C) to uracil (U) in single-stranded DNA 45, 46. Eleven APOBEC (apolipoprotein B mRNA editing catalytic polypeptide-like) proteins, a family of zinc-dependent deaminases, are encoded in the human genome. APOBEC1, APOBEC2, APOBEC3 (with family members A, B, C, D, F, G, and H), APOBEC4, and AID 46, 47. All members of this family have antiviral activity in mammalian cells by inducing lethal editing in the genomes of small DNA viruses, herpesviruses, retroviruses, and RNA viruses such as coronaviruses 46, 48-50. In accordance with this, APOBEC-like directional C to U transitions of genomic plus-strand RNA are substantially overrepresented in COVID-19 pandemic variant SARS-CoV-2 genome sequences 46, 49, 51-53. SARS-CoV-2 sequence data has revealed instances of directional mutation pressures placed on the SARS-CoV-2 genome by host antiviral defense systems 53, 54. The effects of human defense mechanisms like APOBEC on SARS-CoV-2 evolution have been studied extensively 46, 50, 52, 55-57. Based on these studies and the previously established role of AID in B cell tolerance and antibody maturation, we have shown in **Figures 1 & 2**, a possible mechanism for the humoral immune response against SARS-CoV-2 infection 54, 58. We understand that the generation of high affinity antibodies in COVID-19 individual has far reaching consequences with regard to new therapeutic targets and vaccine design. As we are writing this perspective, drug companies have successfully rolled out vaccines and millions have been vaccinated, but researchers are still debating whether new variants could undercut the effectiveness of these vaccines. While SARS-CoV-2 variants are emerging, they pose new challenges for the drug industries and the health care system. Delineating immune mechanisms and identifying novel targets to counter the coronavirus onslaught is very critical 59. It is worth acknowledging that we are moving towards herd immunity, which will potentially be a transition to perpetual immunity, but in the meantime, in addition to robust vaccination, a desperate need to have more options available for the treatment of COVID-19 is overwhelmingly felt. Last but not the least, the threat of having a SAR-CoV-2 variant(s) that could evade our immunity produced by vaccines and topple our efforts is always open to debate. As long as such ideas exist, we are not off the hook, scientists and researchers must explore new ways to redesign the current vaccines but also new drug targets. Mutant strains identified in Brazil and South Africa have already dampen the effects of antibodies crucial for fending off the infection. The current effective way to combat the threat of new virus strains is to quickly vaccinate people. Before a universal vaccine to combat all or any strain of the virus is discovered, scientists must also try to find new therapeutic targets that could initiate or modify an immune response in a way either to sustain or rehabilitate the wanning immunity. In this article we have brought the focus to the most fundamental aspect of an immune response that is the innate power of antibodies.
